# Burnout and risk factors among anesthesia residents and fellows in a conflict-affected context: A national cross-sectional survey

**DOI:** 10.1371/journal.pone.0322940

**Published:** 2025-05-09

**Authors:** Vanda Yazbeck Karam, Zeina Akiki, Wael Salame, Georges Assaf, Caroline Chahine, Rony Nawwar, Micheline Boukhalil, Hanane Barakat

**Affiliations:** 1 Lebanese American University (LAU), Gilbert and Rose-Marie Chagoury School of Medicine, Byblos, Lebanon; 2 Department of Anesthesiology, Lebanese American University (LAU), Gilbert and Rose-Marie Chagoury School of Medicine, Byblos, Lebanon; 3 INSPECT-LB (Institut National de Santé Publique, d’Épidémiologie Clinique et de Toxicologie-Liban), Beirut, Lebanon; 4 Department of Internal Medicine, Psychiatry division, Lebanese American University (LAU), Gilbert and Rose-Marie Chagoury School of Medicine, Byblos, Lebanon; University College Dublin, IRELAND

## Abstract

**Background:**

Burnout is an occupational hazard caused by chronic exposure to excessive work-related stress, negatively impacting both clinicians’ well-being and patient safety**.** Anesthesiology is particularly demanding, and this stress is further exacerbated in regions affected by conflict, where residents and fellows are confronted with additional stressors beyond the usual challenges of medical training. This study aims to assess the proportion and predictors of burnout among anesthesia residents and fellows in Lebanon, a conflict-affected context, by identifying specific drivers of burnout in this population, while also evaluating their association with sociodemographic characteristics.

**Methods:**

A cross-sectional study involving electronic, voluntary, and anonymous survey was sent to all Lebanese anesthesiology residents and fellows at all levels of training, between May and October 2024. The survey utilized the Copenhagen Burnout Inventory (CBI) in addition to other questions. Bivariate and multivariable analyses identified predictors of CBI subscales (personal, work-related, and client-related burnout respectively). An alpha of 0.05 was used to determine statistical significance.

**Results:**

Approximately 71% of participants reported personal burnout, with 32% classified as having a high level. Additionally, 68% reported work-related burnout, with 22% in the high category, and 36% experienced client-related burnout, with 5% classified as high. Moreover, experiencing mental health problems, reporting the need for pharmaceutical or psychological assistance, living with family and covering night shifts were found to be significantly and positively associated with different burnout dimensions.

**Conclusion:**

Burnout levels among anesthesiology residents in conflict-affected areas appear elevated in reference to international studies, which is concerning given the additional stressors associated with ongoing regional conflict. The continuous escalation of these challenges is likely to exacerbate burnout over time. Targeted interventions to manage burnout are crucial for trainees’ well-being and the effective functioning of medical institutions, particularly in conflict-affected regions where the stressors are compounded.

## Introduction

Burnout is an increasingly recognized occupational health concern, especially in high-stress professions like medicine [1,2]. Defined by the World Health Organization (WHO) as a work-related phenomenon and a syndrome characterized by emotional exhaustion, depersonalization, and a reduced sense of professional efficacy, burnout can have severe physical and psychological consequences [2]. First described by Freudenberger in the 1970s [3], burnout typically affects individuals who are highly dedicated to their work, leading to emotional depletion and a gradual loss of motivation. Burnout can also spread within a work environment, negatively impacting colleagues by disrupting team dynamics and performance [4].

In medicine, burnout is particularly common due to the demanding and often high-stakes nature of clinical practice [5,6]. A systematic review conducted in 2021 reported a pooled prevalence of burnout among medical trainees from different specialties of 47.3% (95% confidence interval 43.1% to 51.5%) based on studies published 20 years prior to 2018 [7]. More specifically, a previous study has shown that burnout affects over 50% of anesthesiology residents due to the combined challenges of their medical training and postgraduate examinations [8]. An updated national survey conducted among United States anesthesiology residents reported that up to 24% of residents are at high risk of burnout and 15% experience depression [9]. Moreover, burnout tends to be higher among anesthesiology residents who work extensive hours, and those experiencing depression [10,11]. These rates are concerning, especially that anesthesiologists are also more likely to face issues like substance abuse. In Finland, 25% of anesthesiologists died by suicide, while 22% experienced suicidal ideation. Additionally, anesthesiologists face a higher risk of suicide compared to other specialties [12].

The consequences of burnout extend beyond individual well-being; they also have serious implications for patient safety and quality of care [13,14]. Anesthesiology, in particular, is considered one of the most stressful medical disciplines, with anesthesiologists facing intense responsibilities and managing life-threatening situations [15]. Overnight shifts, weekend work, and high-risk-decision-making further contribute to the stress. The COVID-19 pandemic has intensified these pressures, placing anesthesiologists on the front lines of critical care [16].

Stress and burnout are further exacerbated in regions affected by conflict, where medical trainees face additional pressures. Lebanon has recently fallen into one of the most severe crises in its history, compounded by the COVID-19 pandemic and its impact on the population’s mental health. In addition, the country faces an economic collapse, currency devaluation, the devastating Beirut port exposure in 2020, and a near-collapse of the healthcare sector, intensified by drug shortages and the emigration of skilled healthcare professionals [17,18]. Most recently, bombings in Beirut and various regions since September 2024 due to regional conflict, causing widespread destruction of homes and infrastructure, has heightened the already high level of stress among Lebanese healthcare workers, particularly postgraduate medical trainees, many of whom have lost family members, been forced to leave their homes, or been relocated to safer areas. A 2013 study conducted in Lebanon reported that 27% of residents met the criteria for burnout [19]. A more recent study in 2022 using the Copenhagen Burnout Inventory revealed that 68.6% of residents were experiencing personal burnout, 63.3% work-related burnout, and 35.1% client-related burnout [11]. While there is growing evidence of burnout among Lebanese residents in general, research specifically targeting anesthesiology trainees is limited. This study aims to assess the proportion and associated risk factors of burnout among anesthesia trainees in Lebanon, a conflict-affected region, focusing on the specific drivers of burnout and their association with sociodemographic characteristics.

## Methods

### Study design, settings, and population

A cross-sectional study took place between May and October 2024. The target population was the anesthesiology residents and fellows in training in 2024, at all levels of training from the eight Lebanese Anesthesiology residency programs. Notably, three of these programs offer additional intensive care training alongside anesthesiology. The years of training are from Post Graduate Year (PGY) 1 until PGY5.

### Sample size calculation

The database of the Lebanese Society of Anesthesiologists includes a total of 96 anesthesiology residents and fellows. A previous study on burnout among anesthesiology residents conducted in US, reported that 50% of residents met the criteria for burnout [20]. Using Epi info software with a margin of error of 5% and a confidence interval of 90%, a minimum sample size of 71 participants was determined to be necessary.

### Data collection and ethics statement

An anonymous web-based questionnaire was developed in English using Google Forms and pilot-tested among 5 anesthesiologists and 5 residents. The final questionnaire was distributed to the trainees via email. The study’s scope and purpose were clearly outlined at the beginning of the questionnaire. Participants were informed that their participation was voluntary, with assurances of anonymity and confidentiality. The survey was conducted online and was approved by the Lebanese American University (LAU) IRB (IRB tracking number: LAU.SOM.VA2.21/May/2024). The informed consent procedure was as such: Clicking on the survey link directs participants to an informed consent page, followed by an option to agree. Selecting “yes” indicates the willingness of the participant to continue to the survey questions, and selecting “no” closes the survey. The data collection occurred between May 8th, 2024 and October 3rd, 2024 (database in the Supplementary “S1_File”).

### Measurement tools

The questionnaire was divided into 2 sections. Section 1 focused on personal and occupational risk factors for burnout, along with demographic and practice-related questions, and section 2 assessed burnout levels using the Copenhagen Burnout Inventory (CBI) (questionnaire in the Supplementary “S2_File”).

#### Description of the CBI.

The core concept of burnout in the CBI is fatigue and exhaustion, aligning with the historical development of burnout theory and recent definitions by leading researchers [21]*.* The CBI is a validated tool consisting of three subdimensions: personal burnout, work-related burnout, and client-related burnout. Each scale is intended to be assess burnout in a specific domain.

Personal burnout questions measure a general state of fatigue and exhaustion, applicable to anyone. Work-related burnout questions are intended for individuals with paid work, assessing the degree of burnout perceived to be linked to their job. Client-related burnout questions measure burnout perceived to working with clients (patients, students, children, etc.). In this study, “client” referred to “patient” to fit the context of healthcare.

The CBI includes 19 questions distributed across the three subscales: 6 questions for personal burnout, 7 questions for work-related burnout, and 6 questions for client-related burnout. Responses are scored on a five-point Likert scale, with each item is scored from 0 to 100 (0 = never/almost never or to a very low degree, 25 = seldom or to a low degree, 50 = sometimes or somewhat, 75 = often or to a high degree, 100 = always or to a very high degree). Although the authors of the CBI do not recommend using cutoff scores [21], burnout levels have been categorized in several studies as < versus ≥ 50 [11,22].

### Statistical analysis

The CBI subscales were computed. Descriptive statistics were performed to represent the participants’ characteristics and the CBI subscales, and were expressed as mean ± standard deviation (SD) or median ± interquartile range when appropriate for continuous variables and percentages for qualitative variables. The Cronbach’s alpha coefficients were calculated for the CBI subscales to evaluate internal consistency and the correlations within the subscales were evaluated.

The normality of quantitative variables was assessed using the Shapiro-Wilk test, complemented by visual inspection of histograms and Q-Q plots. For the bivariate analysis, the student’s t-test was used to compare means between two groups, while ANOVA was applied for comparisons involving three or more groups, after verifying homogeneity of variances using Levene’s test. For subgroups with a sample size smaller than 30, or when continuous variables were not normally distributed, non-parametric tests such as the Mann-Whitney U test or the Kruskal-Wallis test were applied. Additionally, the Spearman correlation coefficient was used to examine the relationship between two continuous variables where the assumptions of normality or linearity were not met.

Multivariable analyses were used to evaluate the association between the subscales and the potential confounders after verifying the assumptions of normality of the dependent variables or of the residuals, linearity, homoscedasticity, and absence of multicollinearity. Three multivariable linear regression models were conducted for burnout to identify factors associated with each subscale. The dependent variables were the personal, work-related, and client-related burnout respectively. The variables having a P-value less than 0.05 in the bivariate analysis (such as mental health problems, physical activity…) were included as covariates. Regression coefficients (β) and their 95% confidence intervals (CIs) were reported to quantify the associations between the variables and the subscales.

An alpha of 0.05 was used to determine statistical significance. All analyses were performed using the IBM’s Statistical Package for the Social Sciences (SPSS) version 29.

## Results

### Participants’ characteristics

A total of 74 Anesthesiology trainees participated in this survey with a 77% response rate. The median age was 28 years, 60% being female, and 62% single. Of the participants, 39% were exercising at least once weekly and the majority were non-smokers (85%). Around 20% reported experiencing mental health problems such as depression or anxiety as diagnosed by a physician. In addition, 22% indicated a need for pharmaceutical or psychological assistance “Table 1”.

**Table 1:** Participants’ characteristics and lifestyle habits (n = 74).

		n (%)
**Gender**	Female	44 (60%)
	Male	29 (39%)
	Other	1 (1%)
**Age; Median (Q1; Q3)**		28 (26; 28)
**Minimum–Maximum**		24–32
**Relationship status**	Single	46 (62%)
	In a relationship	26 (35%)
	Others	2 (3%)
Living arrangements*, *Yes %*	Alone	24 (32%)
	Family	47 (64%)
	Others**	17 (23%)
**Physical activity per week**	No/occasionally	45 (61%)
	At least once per week	29 (39%)
**Smoking status**	Non-smokers	63 (85%)
	Smokers	9 (12%)
	Ex-smokers	2 (3%)
**Mental health problems** ^#^	Yes %	14 (19%)
**Need pharmaceutical or psychological assistance**	Yes %	16 (22%)

*Modalities may not add up to 100% because participants may have selected more than one option; ** including hospital residence or shared flat; ^#^as diagnosed by a physician and including depression or anxiety among others; Q1, 1st quartile; Q3, 3rd quartile; All results are expressed as n (%), otherwise specified.

### The practice environment

The highest proportion of participants were affiliated with the first-year residency program (31%). Approximately half reported working 51–60 hours per week, and 66% took 22–30 vacation days per year. The majority worked night shifts (96%), and 76% applied safety rest after night shifts, either consistently or occasionally “Table 2”.

**Table 2:** Participants’ practice environment (n = 74).

		n (%)
**Residency year**	First	23 (31%)
	Second	17 (23%)
	Third	16 (21.5%)
	Fourth	16 (21.5%)
	Fifth	2 (3%)
**Working hours per week**	≤ 50^#^	13 (18%)
	51–60	38 (51%)
	> 60	23 (31%)
**Covering night shifts**	Yes%	71 (96%)
**Application of safety rest after night shift (systemic or not)**	Yes%	56 (76%)
**Number of days’ vacation per year**	7–14	9 (12%)
	15–21	16 (22%)
	22–30	49 (66%)

^#^Of these, 5% work 40 hours or fewer per week

### Participants’ concerns

**Fig 1:**
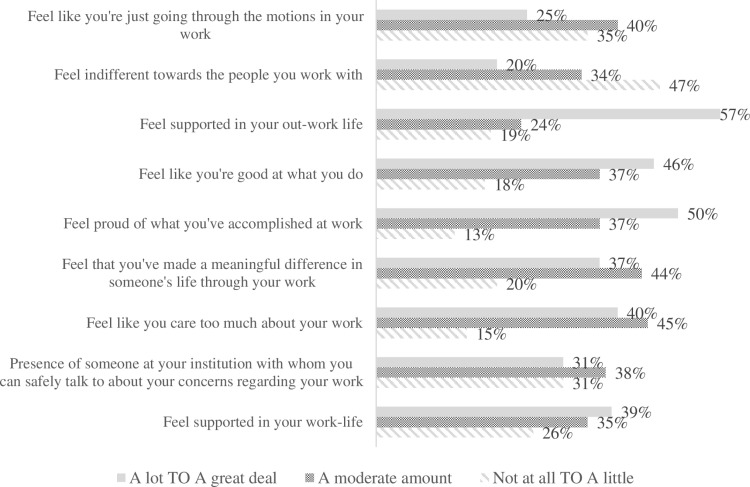
Participants’ concerns.

Almost 40% of the participants reported a strong feeling of being supported in their work-life. Half of them expressed a strong sense of pride in their work accomplishments and 40% stated feeling like they care too much about their work. A smaller proportion reported feeling indifferent towards the people they work with or firmly going through the motions in their work (20% and 25% respectively) “Fig 1”.

### Burnout and CBI subscales

**Fig 2:**
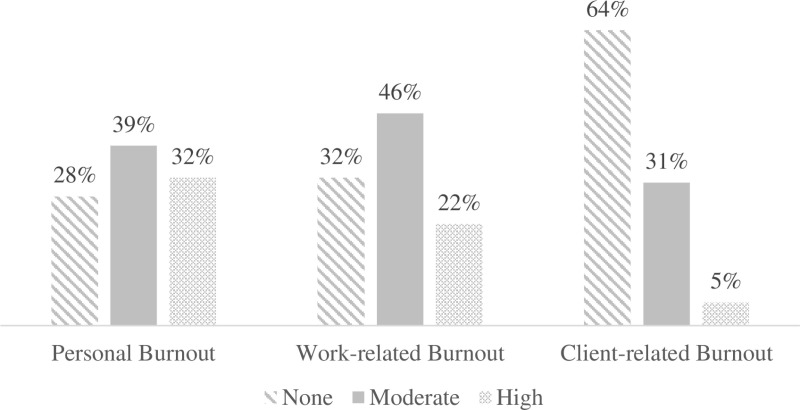
CBI subscales (personal, work-related and client-related respectively) **CBI, Copenhagen Burnout Inventory**.

The Cronbach’s alpha coefficients for the CBI subscales were found to be of 0.92, 0.87 and 0.89 for personal, work-related and client-related burnout respectively, indicating good to excellent internal consistency. In addition, the proportion of burnout was found to be 71%, 68% and 36% for personal (Mean± SD = 58.9 ± 19.6), work-related (Mean± SD = 57.8 ± 18.9) and client-related burnout (Mean± SD = 41.3 ± 18.1) respectively. High levels were found to be of 32% (95% CI: 22% - 44%), 22% (13% - 33%) and 5% (2% - 13%) for personal, work and client-related burnout respectively. A strong positive correlation was observed between personal and work-related burnout and a moderate positive correlation between “personal and client” or “work and client” related burnout levels “Fig 2 and Table 3”.

**Table 3:** CBI subscales.

	Personal Burnout	Work-related burnout	Client-related Burnout
Mean ± SD	59.8 ± 19.6	57.8 ± 18.9	41.3 ± 18.1
Median (Q1; Q3)	62.5 (40.6; 75)	58.9 (42.9; 71.4)	37.5 (29.2; 54.2)
Minimum - Maximum	20.8–100	14.3–100	0–83.3
***Correlation between subscales***			
	**Personal** **Burnout**	**Work-related burnout**	**Client-related Burnout**
Personal burnout		ρ = 0.86 P-value < 0.001	ρ = 0.48 P-value < 0.001
Work-related burnout			ρ = 0.46 P-value < 0.001

### Predictors associated with CBI subscales

#### Personal burnout.

In the bivariate analysis, significant associations were observed with living with family, physical activity, experiencing mental health problems, reporting a need for pharmaceutical or psychological assistance, and number of working hours per week “Table 4” and “S3_File”. The multiple linear regression analysis showed that living with family (Adjusted ß (95% CI) =14.5 (6.3; 22.6)) and experiencing mental health problems (17.6 (6.8; 28.4)) were positively and significantly associated with personal burnout “Table 5 – Model 1”.

**Table 4:** Bivariate analysis for CBI subscales.

		Personal Burnout		Work-related Burnout		Client-related Burnout	
**(Mean ± SD) or ρ**	**P-value**	**(Mean ± SD) or ρ**	**P-value**	**(Mean ± SD) or ρ**	**P-value**
**Gender**	Female	60.7 ± 18.7	0.8	58.7 ± 18.1	0.8	41.5 ± 18.2	
	Male	58.3 ± 21.4		56.8 ± 20.7		39.9 ± 17.3	0.2
	Other	66.7		50		75	
**Age**		−0.15	0.2	−0.09	0.5	0.09	0.7
**Relationship status**	Single	60.8 ± 20.1	0.8	56.5 ± 19.2	0.5	40.9 ± 17.4	0.9
	In a relationship	58.8 ± 19		60.9 ± 18.9		41.8 ± 19.8	
	Others	52.1 ± 26.5		48.2 ± 12.6		45.8 ± 17.7	
**Living arrangements – alone**	Yes	56.6 ± 20.7	0.3	60 ± 20.1	0.5	41.7 ± 16.8	0.9
	No	61.4 ± 19		56.8 ± 18.5		41.2 ± 18.8	
**Living arrangements – family**	Yes	**63.6 ± 18.9**	**0.03**	**61.3 ± 18.7**	**0.04**	41.9 ± 20.3	0.7
	No	**53.4 ± 19.3**		**51.7 ± 18.1**		40.3 ± 13.6	
**Living arrangements – others**	Yes	58.6 ± 18.8	0.7	51.3 ± 18.9	0.1	37.3 ± 11.5	0.3
	No	60.2 ± 20		59.8 ± 18.7		42.5 ± 19.5	
**Physical activity/per week**	Yes	**50 ± 17**	**<0.001**	**49.3 ± 18.1**	**0.001**	**34.8 ± 16.6**	**0.01**
	No/occasionally	**66.2 ± 18.7**		**63.3 ± 17.5**		**45.6 ± 17.9**	
**Smoking status**	Non-smokers	58.7 ± 19.3	0.4	55.9 ± 18.6	0.1	40.5 ± 18.2	0.6
	Smokers	64.8 ± 22.7		66.7 ± 17.8		46.3 ± 17.5	
	Ex-smokers	72.9 ± 14.7		76.8 ± 22.7		45.8 ± 23.6	
**Mental Health problems**	Yes	**70.5 ± 19.4**	**0.02**	**67.6 ± 13**	**0.03**	48.8 ± 19	0.09
	No	**57.4 ± 18.9**		**55.5 ± 19.4**		39.6 ± 17.5	
Need assistance*	Yes	**71.4 ± 18.6**	**0.008**	**72.3 ± 14.7**	**<0.001**	**56.5 ± 20**	**<0.001**
	No	**56.9 ± 18.9**		**53.4 ± 18**		**36.7 ± 14.9**	
**Residency year**	First	66.7 ± 17.3	0.2	61.3 ± 18.6	0.5	40.8 ± 20.2	0.6
	Second	59.6 ± 14.9		59 ± 16.6		46.3 ± 18.7	
	Third	59.4 ± 24.6		58.7 ± 23.4		38.5 ± 15.6	
	Fourth	52.9 ± 20.4		52.7 ± 17.5		41.1 ± 17.7	
	Fifth	43.8 ± 14.7		41.1 ± 2.5		29.2 ± 5.89	
**Working hours per week**	≤ 50	**57.4 ± 17.2**	**0.006**	**52.7 ± 17.8**	**0.02**	**37.5 ± 14.3**	**0.04**
	51–60	**54.4 ± 19.9**		**54 ± 19.1**		**37.9 ± 18.7**	
	> 60	**70.3 ± 16.5**		**66.9 ± 16.6**		**49.1 ± 17**	
**Covering night shifts**	Yes	59.8 ± 20	0.9	57.6 ± 19	0.7	**42.5 ± 17.4**	**0.006**
	No	61.1 ± 6.4		61.9 ± 13.5		**13.9 ± 10.5**	
**Application of safety rest** ^&^	Yes	58.2 ± 19.8	0.2	**54.9 ± 18.8**	**0.02**	41.4 ± 17.9	0.9
	No	65 ± 18.2		**66.9 ± 16.8**		41.2 ± 19	
**Vacation days per year**	7–14	53.7 ± 20.4	0.4	55.2 ± 14.8	0.6	35.6 ± 19.6	0.6
	15–21	64.6 ± 11.9		61.6 ± 15.3		43.8 ± 15.1	
	≥ 22	59.4 ± 21.3		57.1 ± 20.7		41.6 ± 18.8	

*Pharmaceutical or psychological assistance; ^&^after night shifts; SD, Standard Deviation; ρ, Spearman correlation coefficient; Significant associations with P-values < 0.05 are highlighted in bold.

**Table 5:** Multivariable analysis for CBI subscales.

Model 1. Personal burnout		Adjusted ß (95% CI)	P-value
**Living arrangements - family**	Yes	14.5 (6.3; 22.6)	**<0.001**
	No	Reference	
**Mental health problems** ^#^	Yes	17.6 (6.8; 28.4)	**0.002**
	No	Reference	
*R*^*2*^* = 40%; Adjusted for physical activity, need assistance, and working hours per week;* ^#^*such as depression or anxiety*			
**Model 2. Work-related burnout**		**Adjusted ß (95% CI)**	**P-value**
**Living arrangements - family**	Yes	11.5 (3.4; 19.6)	**0.006**
	No	Reference	
**Mental health problems** ^#^	Yes	12 (1.22; 22.7)	**0.03**
	No	Reference	
Need assistance*	Yes	11.2 (1.43; 20)	**0.03**
	No	Reference	
*R*^*2*^* = 38%; Adjusted for physical activity, working hours per week, and safety rest after night shifts;* ^#^*such as depression or anxiety;* *pharmaceutical or psychological assistance			
**Model 3. Client-related burnout**		**Adjusted ß (95% CI)**	**P-value**
Need assistance*	Yes	17.8 (8.5; 27.2)	**<0.001**
	No	Reference	
**Covering night shifts**	Yes	28.5 (10.2; 46.8)	**0.003**
	No	Reference	
*R*^*2*^* = 38%; Adjusted for physical activity, and working hours per week;* *pharmaceutical or psychological assistance			

#### Work-related burnout.

In the bivariate analysis, significant associations were observed with living with family, physical activity, experiencing mental health problems, reporting a need for pharmaceutical or psychological assistance, the number of working hours per week, and the application of safety rest after night shifts (“Table 4” and “S3_File”. The multiple linear regression analysis showed that living with family (11.5 (3.4; 19.6)), experiencing mental health problems (12 (1.22; 22.7)), and reporting the need for pharmaceutical or psychological assistance (11.2 (1.43; 20)) were significantly and positively associated with work-related burnout “Table 5 – Model 2”.

#### Client-related burnout.

In the bivariate analysis, significant associations were observed with physical activity, reporting a need for pharmaceutical or psychological assistance, working hours per week, and night shifts “Table 4” and “S3_File”. The multiple linear regression analysis showed that reporting the need for pharmaceutical or psychological assistance (17.8 (8.5; 27.2)) and taking night shifts (28.5 (10.2; 46.8)) were positively and significantly associated with client-related burnout “Table 5 – Model 3”.

A sub-group analysis was conducted among first-year residents to identify the predictors of personal, work- and client-related burnout. The results are presented in the Supplementary “S4_File”.

## Discussion

This first Lebanese national survey underscores the significant proportion of burnout among anesthesiology trainees in Lebanon, a conflict-affected country, with higher levels of burnout observed in personal and work-related dimensions compared to client-related burnout. Approximately 71% of participants reported personal burnout, with 32% classified as having a high level. Additionally, 68% reported work-related burnout, with 22% in the high category and 36% experienced client-related burnout, with 5% classified as high. Moreover, experiencing mental health problems, reporting the need for pharmaceutical or psychological assistance, living with family and covering night shifts were found to be significantly and positively associated with different burnout dimensions.

In this study, burnout levels among anesthesiology trainees seems to be widespread compared to studies conducted in US. A national study conducted in US in 2023, comparing burnout trends among anesthesiology residents to 2013, reported a decline in the prevalence of high burnout levels, dropping from 41% till 23%. The authors suggested that these reductions in burnout risk and depression may be linked to the implementation of the 2014 Accreditation Council for Graduate Medical Education (ACGME) recommendations which emphasized professionalism and focus on maintaining trainees’ well-being as crucial for developing competent and resilient physicians [9]. Moreover, a 2022 systematic review to assess the prevalence and stressors of burnout in anesthesiology residents, in which the Maslach burnout inventory or its abbreviated form were used, reported that burnout prevalence ranges widely from 2.7 to 67%. Although the reported prevalence of burnout among anesthesiology residents varies across different studies, which could be mainly due to methodological heterogeneity, country of residence, and ongoing collective stressors [10], our findings indicate that burnout levels are quite high and concerning. The heightened levels of stress, insecurity, and resource scarcity in these settings exacerbate burnout, especially among frontline workers like medical trainees [23]. Our results are in line with a 2022 study assessing burnout among PGMT in Lebanon and using CBI where high levels of burnout were reported, with 68.6% for personal burnout, 63.3% for work-related burnout, and 35.1% for client-related burnout [11]. Another study conducted among Lebanese post-graduate trainees using the Oldenburg Burnout Inventory (OLB) reported a 51.1% of exhaustion [24]. This issue could potentially worsen as the ongoing conflict and crisis in the region become prolonged and accumulate over time. The persistence of high burnout levels among anesthesia residents poses long-term risks for Lebanon’s already fragile healthcare system. As more medical professionals seek opportunities abroad or exit the profession due to burnout, the healthcare workforce will shrink, further compromising patient care. Similar findings have been observed in conflict-affected regions where the healthcare systems have faced near collapse. In Syria, burnout rates among medical residents reached 93.75%, with many healthcare providers operating in conditions of extreme psychological and physical distress due to the destruction of medical infrastructure and high casualty rates [25]. Moreover, a 2022 study conducted among Sudanese medical tarinees reported a 70.7% proportion of emotional exhaustion [26]. In Iraq, nearly half of the healthcare professionals emigrated since 2014, further burdening the remaining workforce with excessive workloads and burnout [27].

Our study found that mental health issues were significant predictors of personal and work-related burnout with around 20% of trainees experiencing mental health problems such as depression or anxiety as diagnosed by a physician. Noting that underreporting may occur due to stigma, reluctance to seek professional help, or lack of formal diagnosis [28], this proportion of mental health problems is in line with previous studies which reported anxiety symptoms in 19.8% and depression symptoms in 7.8% of anesthesia and intensive care residents [29]. In fact, the volume of working hours is a significant factor reported in the literature to be associated with poorer mental health, depression and anxiety [30]. Moreover, a study conducted among Nigerian residents reported a significant positive association between working for more than 50 hours per week and depersonalization [31]. In this study, 31% of residents reported working more than 60 hours per week. The demanding workloads, the lack of work-life balance, and emotional strain from patient care responsibilities, might predispose residents to mental health problems and subsequently to burnout.

Moreover, the need for pharmaceutical or psychological assistance was significantly associated with work and client-related burnout. Burnout is widespread due to long hours and high emotional demands [7]. As a result, some medical trainees may seek medication to manage symptoms of anxiety, depression, or stress which can help alleviate the psychological distress associated with burnout. However, the need for medication may indicate a more severe level of distress, which can potentially affect the vigilance of anesthesiology residents and may have an impact on their personal health and patient safety. Addressing burnout proactively through supportive measures can help trainees manage their mental health, improve their resilience, and enhance the quality of care they provide.

The association between living with family and higher burnout, particularly in personal and work-related domains, may reflect the additional responsibilities and stressors arising from family obligations during a crisis. Previous studies in anesthesiology have noted that balancing personal and professional life can be challenging, especially for young physicians living with family, as it may limit their ability to decompress away from work stress [32].

Moreover, taking night shifts was positively associated with client-related burnout. Previous studies have reported the workload factors, such as long working hours and frequent night shifts, were strongly linked to burnout in all dimensions [7], underscoring the importance of work-life balance, mental health support, and effective coping mechanisms for anesthesiology trainees’ well-being.

Interestingly, this study found no association between gender or years of training and burnout, noting that female participants reported slightly higher levels of burnout in the personal and work-related domains compared to their male counterparts, although not statistically significant. Results in the literature are contradictory, where some have reported no associations with gender or years of training [7], while others reported female gender as an exposing factor to burnout among medical trainees [11]. A 2021 French national study identified female gender in anesthesiology residents as a significant predictor for burnout, particularly in work-related domains, due to factors like long working hours and intensive care training. In addition, the study found that third and fourth-year medical trainees experience the highest levels of burnout. While burnout remains significant in the final year of training, there is a noted decline in levels compared to the third or fourth year [29]. The association with gender was also reported by a 2022 study conducted in Lebanon to evaluate burnout among medical trainees [11]. We assume that the work environment might be a more significant contributor to burnout than individual characteristics. In Lebanon, socio-cultural dynamics, including the pressure of close family ties, economic strain, and the stigma surrounding mental health, likely contribute to burnout experiences for both male and female trainees. Additionally, the hierarchical nature of the workplace and the added responsibilities from living with family during a national crisis may overshadow potential gender-related differences seen in other studies. Strong and deep-rooted female leadership and visibility in anesthesiology in Lebanon, might also contribute to a more favorable environment for female anesthesiologists, helping to mitigate gender-related differences in burnout [17]. These dynamics suggest that while gender might play a key role in burnout globally, in a conflict-affected country, broader socio-cultural and mental health challenges are likely more influential in shaping burnout among anesthesiology trainees.

### Practical implications

To effectively manage burnout among anesthesiology residents, a combination of personal, organizational, and policy-level strategies can be implemented:

- Personal Level: Prioritizing adequate sleep, regular physical activity, and a balanced diet, seeking social support from family and friends [11].
- Organizational Level: Implementing leadership trainings, proactively screening for mental health problems, providing mental health support and reducing stigma around mental health, promoting self-care practices and healthy lifestyle habits, enhancing social engagement opportunities, integrating self-care education into the curriculum, and ensuring financial support for research and academic conferences [11,33].
- Policy Level: Establishing standardized duty hour regulations and expanding mental health resources to foster a sustainable work environment [34].

These strategies emphasize the need for both individual and systemic reforms to comprehensively address burnout among medical trainees.

### Strengths and limitations

This study is one of the first to evaluate burnout levels among Anesthesiology trainees in the context of the Lebanese economic crisis and regional conflict. These findings may be applicable to other countries experiencing similar conflicts and instability. The small sample size limits the statistical power and may not fully represent the broader population of residents, potentially leading to biased or less accurate conclusions. However, it met the minimum sample size required for adequate statistical analysis. In addition, several limitations must be acknowledged. First, the non-random sampling method could introduce selection bias, though the 77% response rate helps mitigate this concern. Second, the cross-sectional design of the study limits the ability to interpret causal relationships. Third, the data was self-reported, which could introduce an information bias, yet this risk was minimized by using validated instruments such as the CBI. Lastly, data collection occurred between May and October 2024. As a result, some participants (n = 22) completed the survey after the onset of the bombing in Beirut, which may have affected their burnout levels. However, due to the small sample of respondents surveyed after the regional conflict and Beirut bombing, we were unable to perform a sub-group analysis.

## Conclusion

This study found that burnout levels among anesthesiology residents in conflict-affected regions are concerning, especially as the ongoing regional conflict continues to intensify and accumulate over time. Notably, burnout among medical trainees has been associated with an increasing rate of self-reported medical errors, potentially jeopardizing the integrity required to maintain best practice standards. Targeted interventions and strategies to reduce stress and manage burnout, such as promoting mental health support or encouraging self-care practices, are crucial for trainees’ well-being and the effective functioning of medical institutions. Future research with larger and random samples as well as longitudinal approaches and by focusing on first-year medical trainees are needed to further explore how burnout evolves over time, particularly if the regional conflict continues to escalate and identify associated risk factors especially in crisis-affected regions.

## Supporting information

S1 File Database. (XLSX)

S2 File Questionnaire. (PDF)

S3 File The univariate regressions for the burnout levels. (DOCX)

S4 File The bivariate analysis among first-year residents. (DOCX)
