# Supplementary material for: Burnout and risk factors among anesthesia residents and fellows in a conflict-affected context: A national cross-sectional survey
Source: PLoS One. 2025 May 9;20(5):e0322940. doi: 10.1371/journal.pone.0322940 (PMC12063839; doi:10.1371/journal.pone.0322940)
Supplement: S2 File — (PDF) [file pone.0322940.s002.pdf]

Anesthesiology Residents and Fellows Questionnaire

Dear Anesthesiology Residents and Fellows,
We would like to invite you to participate in a research project by completing the following survey. Our aim is to enhance our comprehension of anesthesiologist-specific risk factors for burnout, particularly within the context of Lebanon.
There are no known risks, harms or discomforts associated with this study beyond those encountered in normal daily life. Completing the survey will take 10 minutes of your time.

By continuing with the survey, you agree with the following statements:

1. *I have been given sufficient information about this research project.*
2. *I understand that my answers will not be released to anyone and my identity will remain anonymous. My name will not be written on the questionnaire nor be kept in any other records.*
3. ***When the results of the study are reported, I will not be identified by name or any other information that could be used to infer my identity.** Only researchers will have access to view any data collected during this research however data cannot be linked to me.*
4. *I understand that I may withdraw from this research any time I wish and that I have the right to skip any question I don't want to answer.*
5. *I understand that my refusal to participate will not result in any penalty or loss of benefits to which I otherwise am entitled to.*
6. *I have been informed that the research abides by all commonly acknowledged ethical codes and that the research project has been reviewed and approved by the Institutional Review Board at the Lebanese American University*
7. *I understand that if I have any additional questions, I can ask the research team listed below.*
8. *I have read and understood all statements on this form.*
9. *I voluntarily agree to take part in this research project by completing the following survey.*

If you have any questions, you may contact:

Name (PI)	*Phone number*	*Email address*
Vanda Abi Raad	*03976327*	*vanda.abiraad@lau.edu.lb*
Hanane Barakat	*03-612009*	*hanane.barakat@lau.edu.lb*

If you have any questions about your rights as a participant in this study, or you want to talk to someone outside the research, please contact the:
Institutional Review Board Office,
Lebanese American University
3rd Floor, Dorm A, Byblos Campus
Tel: 00 961 1 786456 ext. (2546)
irb@lau.edu.lb

Consent to participate in the study:
- ○ *Yes - I agree to participate in this online survey.*
- ○ *No – exit survey*

Part I. Your practice environment

Anesthesiology & Intensive care Study year: *

- 1st year
- 2nd year
- 3rd year
- 4th year
- 5th year

Type of ongoing training: *

Anesthesia □ Yes □ No

Intensive care □ Yes □ No

Other (pain, mobile intensive care….) □ Yes □ No

Number of working hours in an average week: *

□ ≤ 40 hours □ 41 to 50 hours □ 51 to 60 hours □ More than 60 hours

Average number of night shift per month: *

□ 1 to 2 □ 3 to 4 □ 5 to 6 □ More than 6 □ I don't take night shifts

Application of safety rest after night shift: *

□ Yes □ No □ Not systematic

Number of days' vacation per year: *

□ < 7 days □ 7 to 14 days □ 15 to 21 days □ 22 days or more

Do you have someone you currently identify as professional mentor? *

□ Yes □ No

For the following questions, select the most appropriate answer from "Not at all" till "a great deal". *

	**Not at all**	**A little**	**A moderate amount**	**A lot**	**A great deal**
How supported do you feel in your work-life?					
How often is there someone available at your institution with whom you can safely					

talk to about your concerns regarding your work?					
How often do you feel indifferent towards the people you work with?					
How often do you feel like you're just going through the motions in your work?					
How often do you feel like you care too much about your work?					
How often do you feel that you've made a meaningful difference in someone's life through your work?					
How often do you feel proud of what you've accomplished at work?					
How often do you feel like you're good at what you do?					
How supported do you feel in your out-work life?					

Part II - Professional Wellness Evaluation - Copenhagen Burnout Inventory (CBI) *

	Always	Often	Sometimes	Seldom	Never/almost never
How often do you feel tired?					
How often are you physically exhausted?					

How often are you emotionally exhausted?					
How often do you think: “I can’t take it anymore”?					
How often do you feel worn out?					
How often do you feel weak and susceptible to illness?					

	**To a very high degree**	**To a high degree**	**Somewhat**	**To a low degree**	**To a very low degree**
Is your work emotionally exhausting?					
Do you feel burnt out because of your work?					
Does your work frustrate you?					

	**Always**	**Often**	**Sometimes**	**Seldom**	**Never/almost never**
Do you feel worn out at the end of the working day?					
Are you exhausted in the morning at the thought of another day at work?					
Do you feel that every working hour is tiring for you?					
Do you have enough energy for family and friends during leisure time?					

	To a very high degree	To a high degree	Somewhat	To a low degree	To a very low degree
Do you find it hard to work with patients?					
Do you find it frustrating to work with patients?					
Does it drain your energy to work with patients?					
Do you feel that you give more than you get back when you work with patients?					

	Always	Often	Sometimes	Seldom	Never/almost never
Are you tired of working with patients?					
Do you sometimes wonder how long you will be able to continue working with patients?					

Part III. Your Health and Lifestyle

Gender? *

- Male
- Female
- Others

Year of birth*: ____________

Marital status: *

- Single
- In a relationship
- Others

Living arrangement: *

- Alone

- Family
- Others (in a shared flat, in a hospital residence...)

Are you a: *

- Current smoker (cigarettes, waterpipes and/or any other type)
- Ex-smoker (have smoked more than 100 cigarettes in your lifetime but have not smoked in the last 28 days OR/AND used to smoke waterpipes regularly at a frequency of at least once a month but have not smoked in more than one month)
- Non-smoker

Are you currently engaged in any type of physical activity? *

- Yes, daily
- 3 to 6 times per week
- 1 to 2 times per week
- No / occasionally

Do you currently have any caregiving responsibilities outside of work? * □ Yes □ No

If yes, for how many people? (Children, older parents….) ____________________________

Do you suffer from any of the following chronic physical health problems? *

Asthma □ Yes □ No

Obesity □ Yes □ No

Others □ Yes □ No

Do you suffer from any of the following chronic mental health problems (as diagnosed by a physician)? *

Depression □ Yes □ No

Anxiety □ Yes □ No

Others □ Yes □ No

Do you need pharmaceutical or psychological assistance? *

□ Yes □ No

Part IV: Additional thoughts

Is there anything else you would like to share or comment on regarding the survey questions:

____

____

Thank you for taking the time to answer this survey!
