# Supplementary material for: Burnout and risk factors among anesthesia residents and fellows in a conflict-affected context: A national cross-sectional survey
Source: PLoS One. 2025 May 9;20(5):e0322940. doi: 10.1371/journal.pone.0322940 (PMC12063839; doi:10.1371/journal.pone.0322940)
Supplement: S3 File — (DOCX) [file pone.0322940.s003.docx]

**Title: *Burnout and Risk Factors Among Anesthesia Residents and Fellows in a Conflict-Affected Context: A National Cross-Sectional Survey***

**Supplementary – S2**

**Table S2. The univariate regressions for the burnout levels**

|  |  | **Personal**  **Burnout** |  | **Work-related Burnout** | |  | **Client-related Burnout** | |  |
| --- | --- | --- | --- | --- | --- | --- | --- | --- | --- |
|  |  | **Unadjusted beta (95% CI)** | **P-value** | **Unadjusted beta (95% CI)** | **P-value** | | **Unadjusted beta (95% CI)** | **P-value** | |
| **Gender** | Female | -2.98 (-22.1; 16.1) | 0.8 | 4.34 (-14.1; 23) | | 0.6 | -16.8 (-35.3; 1.8) | | 0.08 |
|  | Male | -8.33 (-52.8; 36.2) | 0.7 | 6.77 (-36.4; 50) | | 0.8 | -35 (-71; 0.9) | | 0.06 |
|  | Other | Reference |  | Reference | |  | Reference | |  |
| **Age** |  | -1.55 (-4.09; 0.99) | 0.2 | -7.11 (-3.19; 1.77) | | 0.6 | 0.4 (-2; 2.77) | | 0.7 |
| **Relationship status** | Single | -4.35 (-19.1; 10.4) | 0.6 | -4.15 (-18; 9.7) | | 0.6 | 2.5 (-10.2; 15) | | 0.7 |
|  | In a relationship | -6.73 (-35.9; 22.4) | 0.6 | -12.6 (-41; 15.5) | | 0.4 | 4 (-26; 34) | | 0.8 |
|  | Others | Reference |  | Reference | |  | Reference | |  |
| **Living arrangements – alone** | Yes | -4.82 (-14.5; 4.88) | 0.3 | 3.19 (-6.22; 12.6) | | 0.5 | 0.5 (-8.5; 9.5) | | 0.9 |
|  | No | Reference |  | Reference | |  | Reference | |  |
| **Living arrangements – family** | Yes | 10.2 (0.98; 19.4) | **0.03** | 9.60 (0.70; 18.5) | | **0.04** | 1.67 (-7.1; 10.4) | | 0.7 |
|  | No | Reference |  | Reference | |  | Reference | |  |
| **Living arrangements – others** | Yes | -1.66 (-12.5; 9.2) | 0.8 | -8.51 (-18.8; 1.8) | | 0.1 | -5.29 (-15.2; 4.65) | | 0.3 |
|  | No | Reference |  | Reference | |  | Reference | |  |
| **Physical activity/per week** | Yes | -16.2 (-24.8; -7.7) | **<0.001** | -14.1 (-22.5; -5.7) | | **0.001** | -10.8 (-19; -2.53) | | **0.01** |
|  | No/occasionally | Reference |  | Reference | |  | Reference | |  |
| **Smoking status** | Non-smokers | Reference |  | Reference | |  | Reference | |  |
|  | Smokers | 6.09 (-7.91; 20.1) | 0.4 | 10.7 (-2.45; 24) | | 0.1 | 5.82 (-7.05; 18.7) | | 0.4 |
|  | Ex-smokers | 7.09 (-6.69; 20.9) | 0.3 | 10.4 (-3; 23.8) | | 0.1 | 2.68 (-10.4; 15.8) | | 0.7 |
| **Mental Health problems** | Yes | 13.2 (1.92; 24.4) | **0.02** | 12.1 (1.14; 23) | | **0.03** | 9.23 (-1.31; 19.8) | | 0.09 |
|  | No | Reference |  | Reference | |  | Reference | |  |
| **Need assistance*** | Yes | 14.5 (3.86; 25.1) | **0.008** | 18.9 (9.07; 29) | | **<0.001** | 19.8 (10.7; 28.9) | | **<0.001** |
|  | No | Reference |  | Reference | |  | Reference | |  |
| **Residency year** | First | 22.9 (-3.27; 49.1) | 0.08 | 20.3 (-7.6; 48) | | 0.1 | 11.6 (-18.6; 42) | | 0.4 |
|  | Second | 15.8 (-7.62; 39.2) | 0.2 | 18 (-7.4; 43) | | 0.2 | 17.2 (-12; 46) | | 0.2 |
|  | Third | 15.6 (-22.7; 53.9) | 0.4 | 17.6 (-18.5; 54) | | 0.3 | 9.38 (-14.7; 33) | | 0.4 |
|  | Fourth | 9.12 (-22.8; 41.1) | 0.6 | 11.6 (-15.4; 39) | | 0.4 | 12 (-15.3; 39) | | 0.4 |
|  | Fifth | Reference |  | Reference | |  | Reference | |  |
| **Working hours per week** | ≤ 50 | Reference |  | Reference | |  | Reference | |  |
|  | 51 to 60 | -11.2 (-20; -2.5) | **0.01** | -7.8 (-16.4; 0.9) | | 0.08 | -7 (-15.2; 1.3) | | 0.09 |
|  | > 60 | 15.1 (5.93; 24.4) | **0.002** | 13.2 (4.2; 22.2) | | **0.005** | 11.3 (2.56; 20) | | **0.01** |
| **Covering night shifts** | Yes | -1.31 (-24.5; 21.9) | 0.9 | -4.26 (-27; 18.1) | | 0.7 | 28.6 (8.3; 49) | | **0.006** |
|  | No | Reference |  | Reference | |  | Reference | |  |
| **Application of safety rest** | Yes | -6.86 (-17.4; 3.67) | 0.2 | -12 (-22; -2.04) | | **0.02** | 0.17 (-9.7; 10) | | 0.9 |
|  | No | Reference |  | Reference | |  | Reference | |  |
| **Vacation days per year** | 7 to 14 | Reference |  | Reference | |  | Reference | |  |
|  | 15 to 21 | 10.9 (-2.37; 24.1) | 0.1 | 6.45 (-6.6; 19.5) | | 0.3 | 8.1 (-6.35; 23) | | 0.3 |
|  | ≥ 22 | 2.87 (-4.84; 10.6) | 0.5 | 0.96 (-6.3; 8.21) | | 0.8 | 3 (-3.9; 9.8) | | 0.4 |
