# Supplementary material for: Burnout and risk factors among anesthesia residents and fellows in a conflict-affected context: A national cross-sectional survey
Source: PLoS One. 2025 May 9;20(5):e0322940. doi: 10.1371/journal.pone.0322940 (PMC12063839; doi:10.1371/journal.pone.0322940)
Supplement: S4 File — (DOCX) [file pone.0322940.s004.docx]

**Title: *Burnout and Risk Factors Among Anesthesia Residents and Fellows in a Conflict-Affected Context: A National Cross-Sectional Survey***

**Supplementary – S3**

**The bivariate analysis among first-year residents**

The bivariate analyis of burnout levels only among first-year residents is shown in Table S2 below.

Compared to the whole sample, the following associations were observed among first-year residents:

- Age was positively correlated with client-related burnout.
- Similar associations were observed for residents living with their families.
- Residents living in hospital residency or in a shared flat reported significantly lower work-related burnout.
- Physically active residents experienced significantly less personal burnout but not work- or client-related burnout.
- Experiencing mental health problems was significantly and positively associated with client-related burnout rather than personal or work-related burnout.
- The need for assistance was significantly associated with only personal and work-related burnout.
- Working more hours per week was significantly associated only with the client-related burnout.
- Similar associations were observed for covering night shifts.
- No significant associations were observed regarding the application of safety rest after night shifts and work-related burnout.

The multivariable analysis was not feasible due to the small sample among first-year residents

| **Table S2. Bivariate analysis among first-year residents; n=23** | | | | | | | |  |
| --- | --- | --- | --- | --- | --- | --- | --- | --- |
|  |  | **Personal**  **Burnout** | | **Work-related Burnout** | | **Client-related Burnout** | |  |
|  |  | **(Mean ± SD) or ρ** | **P-value** | **(Mean ± SD) or ρ** | **P-value** | **(Mean ± SD) or ρ** | **P-value** | |
| Gender | Female | 69.9 ± 16 | 0.3 | 65.8 ± 18 | 0.2 | 44.3 ± 18.5 | 0.3 | |
|  | Male | 61.6 ± 19 |  | 54.4 ± 19 |  | 35.2 ± 22.6 |  | |
| Age |  | 0.31 | 0.1 | 0.29 | 0.2 | 0.55 | **0.007** | |
| Relationship status | Single | 65.7 ± 17.2 | 0.4 | 60.2 ± 18.1 | 0.4 | 38.7 ± 19.8 | 0.1 | |
|  | In a relationship | 77.1 ± 20.6 |  | 73.2 ± 27.8 |  | 62.5 ± 11.8 |  | |
| Living arrangements – alone | Yes | 66.7 ± 23 | 1 | 66.4 ± 19 | 0.5 | 35.8 ± 21 | 0.5 | |
|  | No | 66.7 ± 16 |  | 59.9 ± 19 |  | 42.1 ± 20 |  | |
| Living arrangements – family | Yes | 71.5 ± 14.7 | **0.007** | 66.9 ± 15.4 | **0.004** | 43.3 ± 22 | 0.3 | |
|  | No | 49.2 ± 15.4 |  | 41.4 ± 16.5 |  | 31.7 ± 8.1 |  | |
| Living arrangements – others | Yes | 55 ± 10.8 | 0.09 | 42.9 ± 16.6 | **0.009** | 33.3 ± 5.9 | 0.4 | |
|  | No | 70 ± 17.5 |  | 66.5 ± 16 |  | 42.8 ± 22,4 |  | |
| Physical activity/ per week | Yes | 54.2 ± 17.1 | **0.008** | 52.2 ± 20.5 | 0.09 | 32.3 ± 17.2 | 0.1 | |
|  | No/occasionally | 73.3 ± 13.6 |  | 66.2 ± 16.2 |  | 45.3 ± 20.8 |  | |
| Smoking status | Non-smokers | 64 ± 17.4 | 0.3 | 57.3 ± 17.1 | 0.08 | 40.8 ± 20.5 | 0.8 | |
|  | Smokers | 77.8 ± 12.7 |  | 76.2 ± 14.4 |  | 44.4 ± 25.1 |  | |
|  | Ex-smokers | 83.3 |  | 92.9 |  | 29.2 |  | |
| Mental Health problems | Yes | 83.3 ± 18.2 | 0.07 | 75 ± 15.6 | 0.2 | 63.9 ± 23.7 | **0.03** | |
|  | No | 64.2 ± 16 |  | 59.3 ± 18.5 |  | 37.3 ± 17.8 |  | |
| Need assistance* | Yes | 79.2 ± 12.4 | **0.04** | 75.6 ± 10.2 | **0.03** | 53.5 ± 27.4 | 0.07 | |
|  | No | 62.3 ± 16.8 |  | 56.3 ± 18.5 |  | 36.3 ± 15.6 |  | |
| Working hours per week | ≤ 50 | 66.7 ±14 | 0.2 | 55 ± 23 | 0.2 | 37.5 ± 13.5 | **0.02** | |
|  | 51 to 60 | 60.6 ± 19.8 |  | 60.7 ± 21.2 |  | 31.8 ± 18.9 |  | |
|  | > 60 | 76.2 ± 11.7 |  | 66.8 ± 10 |  | 57.1 ± 17.6 |  | |
| Covering night shifts | Yes | 67.5 ± 18 | 0.6 | 61.3 ± 19.6 | 0.9 | 44.8 ± 18.2 | **0.01** | |
|  | No | 61.1 ± 6.4 |  | 61.9 ± 13.5 |  | 13.9 ± 10.5 |  | |
| Application of safety rest after night shift | Yes | 65.3 ±19 | 0.5 | 58.1 ± 18.3 | 0.1 | 39.4 ± 21.3 | 0.5 | |
|  | No | 71.7 ± 9 |  | 72.9 ± 16.5 |  | 45.8 ± 16.7 |  | |
| Number of days’ vacation per year | 7 to 14 | 70.8 ± 11.8 | 0.9 | 62.5 ± 2.5 | 0.9 | 47.9 ± 14.7 | 0.6 | |
|  | 15 to 21 | 65.9 ±17.6 |  | 61.3 ± 19.6 |  | 34 ± 11.3 |  | |
|  | ≥ 22 | 66.4 ± 18.6 |  | 61.2 ± 20.2 |  | 42.5 ± 23.5 |  | |
